# Jeziorny Method Should Be Avoided in Avrami Analysis of Nonisothermal Crystallization

**DOI:** 10.3390/polym15010197

**Published:** 2022-12-30

**Authors:** Sergey Vyazovkin

**Affiliations:** Department of Chemistry, University of Alabama at Birmingham, 901 S. 14th Street, Birmingham, AL 35294, USA; vyazovkin@uab.edu

**Keywords:** Avrami, calorimetry, crystallization, kinetics, polymer

## Abstract

The Jeziorny method treats nonisothermal crystallization data by replacing the variable temperature (*T*) values with the corresponding values of time and substituting them into the isothermal Avrami plot, ln[−ln(1 − *α*)] vs. ln*t*. For isothermal data, the slope of this plot is the Avrami exponent, *n* and the intercept is the rate constant, *k_A_*. This does not hold for nonisothermal data. Theoretical analysis suggests that in the case of nonisothermal data the intercept cannot be interpreted as *k_A_*, and its “correction” by dividing over the temperature change rate *β* is devoid of any meaning. In turn, the slope cannot be interpreted as *n*. It is demonstrated that the slope changes with time and its value depends not only on *n* but also on the temperature, temperature range, and activation energy of crystallization. Generally, the value of the slope is likely to markedly exceed the *n* value. The theoretical results are confirmed by analysis of simulated data. Overall, the Jeziorny method as well as other techniques that substitute nonisothermal data into the isothermal Avrami plot should be avoided as invalid and useless for any reasonable Avrami analysis. It is noted that *n* can be estimated from the nonlinear plot of ln[−ln(1 − *α*)] vs. *T*.

## 1. Introduction

Polymer crystallization kinetics under nonisothermal conditions is a topic of great practical interest. The crystallization kinetics are commonly parameterized in terms of the Avrami model (also known as the Johnson–Mehl–Avrami–Erofeev–Kolmogorov model [1,2]). The classical approach to the problem is the use of the Ozawa method [3]. In his seminal work, Ozawa used theoretical considerations to adjust the Avrami model to nonisothermal conditions and to develop a method of estimating the Avrami exponent. The latter is of interest as it can be linked to the dimensionality of the crystal growth. Judging by over 2000 citations [4] of the original publication [3], the Ozawa method appears to be the most popular approach to the Avrami analysis of nonisothermal crystallization. Another very popular (over 1000 citations [4]) approach to the problem is the use of the so-called Jeziorny method [5]. Unlike the Ozawa method, the one by Jeziorny was proposed on entirely empirical grounds. Despite that, it had not been thoroughly tested before Zhang et al. [6], who used simulated data to compare the Ozawa and Jeziorny methods. The results of that study have demonstrated that the Jeziorny method overestimates significantly the Avrami exponent, whereas the Ozawa method estimates the correct value. Most recently, Kourtidou and Chrissafis [7] have used experimental data to test the Jeziorny method. Again, it has been found that compared to other methods it yields significantly overestimated (about four times) values of the Avrami exponent. The latter has been reported [7] to exceed physically meaningful values.

The results of the aforementioned tests certainly raise serious concerns regarding the validity of the Jeziorny method. However, individual examples of the failure are not yet proof of the faulty nature of the method. One can also find examples where the Jeziorny method yields the Avrami exponents that are reasonably consistent with the ones estimated by the Ozawa method [8,9,10]. A proper way to understand the faults of the Jeziorny method is via its theoretical analysis. The purpose of the present article is to provide such analysis.

## 2. Avrami Model

The classical Avrami model applies to isothermal conditions and is typically used in the following form [1,2]:

(1) $$1-\alpha =\exp\left[-k_{A}\left(T\right)t^{n}\right]$$

where *t* is the time, *α* is the fractional volume transformed to the crystalline phase, *n* is the Avrami exponent, *k_A_*(*T*) is the Avrami rate constant. Then, the parameters of the Avrami model can be estimated from Equation (2):

(2) $$\ln\left[-\ln\left(1-\alpha \right)\right]=n\ln t+\ln k_{A}\left(T\right)$$

In practical terms, plotting ln[−ln(1 − *α*)] against ln*t* for a given constant temperature yields a straight line. The slope of this plot yields the Avrami exponent, which is expressed mathematically as the following derivative:

(3) $$\frac{d\ln\left[-\ln\left(1-\alpha \right)\right]\;}{d\ln t}=n$$

In turn, the intercept gives the rate constant at a given temperature.

Note that a nonisothermal form of Equation (1) contains the time integral of *k_A_*(*T*) as found in a paper by Nakamura et al. [11].

## 3. Jeziorny Method

The idea of the Jeziorny method is that Equation (2) can be applied to nonisothermal data by replacing temperature with time and introducing a correction factor for the rate constant. Indeed, one can easily switch from temperature to time as the former is usually varied linearly with the latter so that:

(4) $$t=\frac{T-T_{0}}{\beta }$$

where *T*_0_ is the temperature at which crystallization starts and *β* is the heating rate. However, one must keep in mind that all values of the time determined in such a way correspond to different temperatures. In contrast, the isothermal Equation (2) requires the time values to correspond to the same constant temperature. This, of course, is an important contradiction. To put it differently, the substitution of the time from Equation (4) into the isothermal Avrami equation has no theoretical justification [12].

The correction factor that is supposed to adjust Equation (2) to nonisothermal conditions has the following form [5]:

(5) $$\ln k_{A}^{*}=\frac{\ln k_{A}\left(T\right)}{\beta }$$

As mentioned earlier [13], this correction is fundamentally wrong as it violates the basic concept of equating physical quantities; one can only equate the quantities that have the same units of measurement. The left hand side of Equation (5) has no units, whereas its right hand side has the units of min K^−1^. That is, Equation (5) is as meaningless as the statement that 1 gram equals 1 meter.

The Jeziorny method is employed by plotting ln[−ln(1 − *α*)] against ln*t*. Usually, such a plot is nearly linear so that its slope is interpreted as *n* and intercept as $\ln k_{A}^{*}$, i.e., by analogy with Equation (2). This interpretation is incorrect in the case of nonisothermal data. Apart from the fact that the “corrected” $\ln k_{A}^{*}$ is physically meaningless, it is estimated as a single number from the respective single value of the intercept. This does not make any sense because, as per Equation (5), $\ln k_{A}^{*}$ is defined via the temperature dependent $\ln k_{A}\left(T\right)$ and dividing the latter over *β* does not eliminate the temperature dependence. In other words, for nonisothermal data $\ln k_{A}^{*}$ cannot in principle be represented by a single value because it is necessarily variable.

Furthermore, for nonisothermal data the slope of the ln[−ln(1 − *α*)] vs. ln*t* plot is not *n*. Taking into account the aforementioned modifications, the application of the Jeziorny method boils down to the use of the following equation:

(6) $$\ln\left[-\ln\left(1-\alpha \right)\right]=n\ln\frac{T-T_{0}}{\beta }+\frac{\ln k_{A}\left(T\right)}{\beta }$$

For the reasons explained above, this equation is invalid. Even if it were valid, its slope would not give the value of *n*. By definition, the slope of the ln[−ln(1 − *α*)] vs. ln*t* dependence is determined by the respective derivative. In the first approximation, $\ln k_{A}\left(T\right)$ for crystallization is frequently assumed [14] to have the Arrhenius form:

(7) $$\ln k_{A}\left(T\right)=\ln A-\frac{E}{RT}$$

so that the slope is:

(8) $$\frac{d\ln\left[-\ln\left(1-\alpha \right)\right]\;}{d\ln t}=n+\frac{E}{R}\frac{t}{{\left(T_{0}+\beta t\right)}^{2}}$$

Clearly, the slope is larger than *n* by the value of the second addend. Since the latter is temperature dependent, so is the slope. Additionally, the invalid “correction” via Equation (5) gives rise to the invalid summation in the right hand side of Equation (8). Just as one cannot equate the quantities that have different units of measurement, one cannot add them either. Indeed, *n* is unitless, whereas the second addend has the units of min K^−1^.

Note that without the “correction”, the second addend in Equation (8) has *β* in the numerator:

(9) $$\frac{d\ln\left[-\ln\left(1-\alpha \right)\right]\;}{d\ln t}=n+\frac{E}{R}\frac{\beta t}{{\left(T_{0}+\beta t\right)}^{2}}$$

so that the problem of the invalid summation vanishes. However, eliminating the “correction” cannot make Equation (6) valid because the essence of its invalidity is in applying the isothermal equation Equation (2) to nonisothermal data. A proper equation for the ln[−ln(1 − *α*)] vs. ln*t* slope is derived in the next section.

It should be stressed that the conclusions of this section are relevant not only to the Jeziorny method. They are also relevant to the quite common Avrami analyses [15,16,17,18,19,20,21] that substitute nonisothermal data into the isothermal Avrami plot, which essentially is the Jeziorny method without the “correction factor” (Equation (5)).

## 4. Nonisothermal Form of Avrami Model

To become applicable to nonisothermal data, the Avrami equation has to be converted to a nonisothermal form. This is performed best by starting with a slightly modified isothermal form:

(10) $$1-\alpha =\exp\left[-{\left(k_{mA}\left(T\right)t\right)}^{n}\right]$$

where $k_{mA}\left(T\right)$ is the modified Avrami rate constant. An advantage of this form is that the respective rate constant always has units of time^−1^, i.e., the units of the conversion rate, dα/dt. The resulting $k_{mA}\left(T\right)$ values can be substituted directly into the Arrhenius Equation (7) to estimate the correct values of ln*A* and *E*. On the contrary, $k_{A}\left(T\right)$ values have units of time^−n^ and their direct substitution into the Arrhenius equation yields ln*A* and *E* values that are *n* times larger than the correct ones [22,23,24,25,26]. Note that the given modification changes Equation (2) into:

(11) $$\ln\left[-\ln\left(1-\alpha \right)\right]=n\ln k_{mA}\left(T\right)+n\ln t$$

This, however, does not affect the slope of the ln[−ln(1 − *α*)] vs. ln*t* plot. Per Equation (3), it remains equal to *n*.

Naturally, Equation (10) is still isothermal. As already mentioned, its nonisothermal form includes integration with respect to the time [11]. However, when temperature is varied linearly with time, the time integral is easily replaceable with the temperature integral:

(12) $${\left[-\ln\left(1-\alpha \right)\right]}^{1/n}=\frac{A}{\beta }\int_{T_{0}}^{T}\exp\left(\frac{-E}{RT}\right)dT$$

It should be stressed that the resulting nonisothermal form of the Avrami equation is valid when a system is saturated with the nuclei so that the crystallization rate is limited by the rate of their growth [22,23,27]. It is also valid when the rates of nucleation and growth have the same temperature dependence [28] or, more specifically, the same Arrhenian activation energies [29]. Nevertheless, if the latter condition is not satisfied, Equation (12) acquires a constant multiplier, which means that it still remains applicable [29].

The main difference between Equation (12) and the one used in the Nakamura et al. method, is in the different types of temperature dependencies for *k_A_*(*T*). Equation (12) employs the Arrhenius dependence, whereas the Nakamura et al. method uses the dependence of the Williams–Landel–Ferry type. Both dependencies are approximations that hold for a narrower temperature range. For a broad range, it is more appropriate to use the more complex Hoffman–Lauritzen equation [30]. Yet, the use of the Arrhenius approximation is still adequate for obtaining insights into the meaning of the ln[−ln(1 − *α*)] vs. ln*t* plot.

The temperature integral in Equation (12) does not have an analytical solution. Its integration inevitably involves the use of approximating functions, *p*(*x*):

(13) $$\frac{A}{\beta }\int_{0}^{T}\exp\left(\frac{-E}{RT}\right)dT=\frac{AE}{\beta R}p\left(x\right)$$

where *x* = *E*/*RT*. Equation (13) replaces the lower integration limit in Equation (12) with 0, which simplifies integration without generally diminishing its accuracy [31]. The *p*(*x*) functions are available in a large variety of complexities and accuracies [32]. The following function [33]

(14) $$p\left(x\right)=\frac{\exp\left(-1.0008x-0.312\right)}{x^{1.92}}$$

is one of the most accurate among the simpler ones. Its substitution into Equation (13) followed by the substitution of Equation (13) into (12) yields:

(15) $$\ln\left[-\ln\left(1-\alpha \right)\right]=n\left(\ln\frac{AE}{\beta R}-1.92\ln\frac{E}{R}-0.312\right)+1.92n\ln T-1.0008n\frac{E}{RT}$$

or simply:

(16) $$\ln\left[-\ln\left(1-\alpha \right)\right]=nConst+1.92n\ln T-1.0008n\frac{E}{RT}$$

where *Const* collects all temperature independent terms.

Equation (16) is a nonisothermal form of the Avrami equation. Unlike Equation (2), this equation is suitable for replacing temperature with time. In accord with Equation (4), Equation (16) transforms into an equation with the time dependent terms:

(17) $$\ln\left[-\ln\left(1-\alpha \right)\right]=nConst+1.92n\ln\left(T_{0}+\beta t\right)-1.0008n\frac{E}{R\left(T_{0}+\beta t\right)}$$

The importance of Equation (17) is that it can be used to understand the true meaning of the slope of ln[−ln(1 − *α*)] vs. ln*t* in the case of nonisothermal data. By taking the derivative of Equation (17) with respect to ln*t* one obtains the value of the slope as follows:

(18) $$\frac{d\ln\left[-\ln\left(1-\alpha \right)\right]\;}{d\ln t}=1.92n\frac{\beta t}{T_{0}+\beta t}+1.0008n\frac{E}{R}\frac{\beta t}{{\left(T_{0}+\beta t\right)}^{2}}$$

Equation (18) indicates clearly that when one plots ln[−ln(1 − *α*)] vs. ln*t* the resulting slope does not give the value of the Avrami exponent. It also suggests that the slope is time and, thus, temperature dependent, which means the ln[−ln(1 − *α*)] vs. ln*t* plot is nonlinear. The dependence is quite complex. If the first addend depends on the crystallization temperature (*T*_0_ + *βt*) and the temperature interval of crystallization (*βt*), the second also depends on the activation energy of crystallization. Furthermore, the second addend is likely to be markedly larger than the first one. For typical crystallization conditions [7,34,35], *βt* is roughly around 40 K, whereas *T*_0_ + *βt* is around 400 K. This makes the first addend around 0.2*n*. For the same temperature conditions, the second addend is roughly around 3*n* for *E* = 100 kJ mol^−1^ or *n* for 30 kJ mol^−1^. In either case, the sum of these two addends exceeds the actual value *n*. The fact that the Jeziorny method yields excessive values of the Avrami exponent has already been noted [6,7]. However, depending on the crystallization parameters (lower activation energy, higher temperature, or narrower temperature range) the second addend can also possibly drop below *n* so that the slope can, in principle, yield the values around or even smaller than *n*. It means that the ln[−ln(1 − *α*)] vs. ln*t* plot may occasionally yield the Avrami exponent, which can explain why the Jeziorny method sometimes evaluates *n* values that are reasonably consistent with those determined by the Ozawa method. However, this cannot occur as the general case.

It is noteworthy that Equation (15) can be easily linked to the Ozawa method [3]. This method uses isothermal sections of nonisothermal data so that the Avrami exponent is determined as the slope of the ln[−ln(1 − α)] vs. ln*β* plot. In Equation (15) only one term depends on *β*, and the temperature dependent terms are constant for isothermal sections. As a result, the respective derivative is:

(19) $$\frac{d\ln\left[-\ln\left(1-\alpha \right)\right]\;}{d\ln\beta }=-n$$

That is, Equation (15) gives exactly the same result as the Ozawa method.

## 5. Simulations

Theoretical analysis in the previous section has permitted the obtaining of an equation for the slope of the ln[−ln(1 − *α*)] vs. ln*t* plot. In this section, the obtained result is tested by using simulated data that correspond to the Avrami process with *n* = 3. The process was simulated assuming the Arrhenius temperature dependence with *E* = 100 kJ mol^−1^ and *A* = 10^12^ min^−1^, for the heating rates 1, 1.5, 2, 3, and 5 K min^−1^. The complete details of the simulations are presented elsewhere [36].

**Figure 1:**
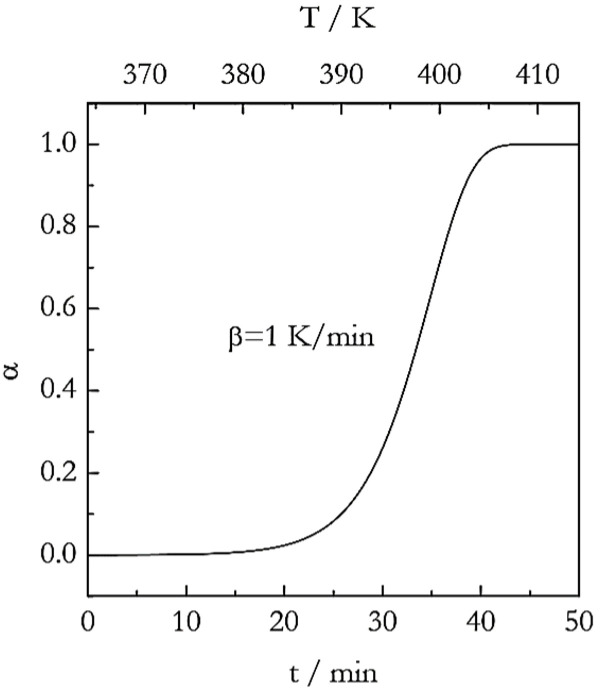
Simulated nonisothermal data for a process with the Avrami exponent, *n* = 3.

Figure 1 displays the simulated data at 1 K min^−1^. The curve was simulated in the form α vs. *T* and then converted into the form α vs. *t* in accord with Equation (4). It is seen that the curve remains the same whether it is plotted against temperature or time. In other words, the use of Equation (4) does not change the nature of the data, which remains invariably nonisothermal.

**Figure 2:**
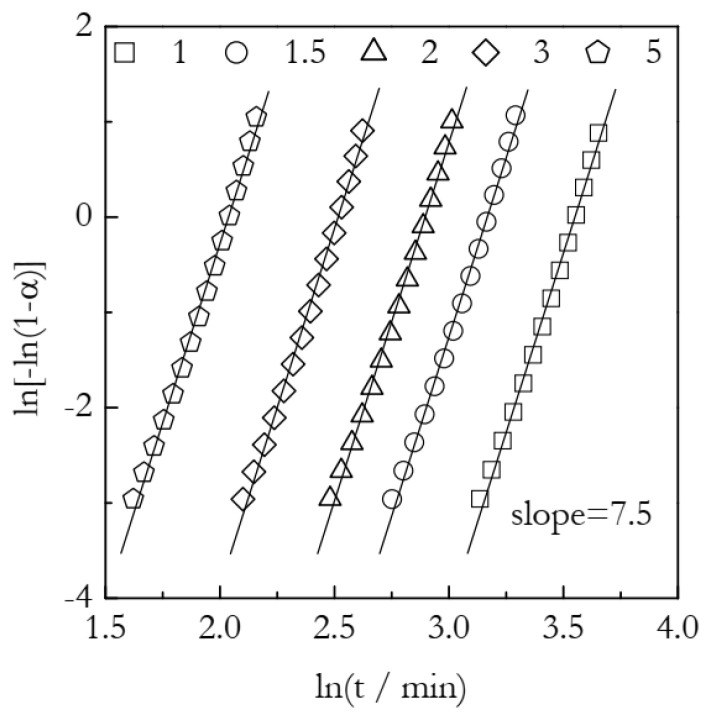
Avrami plots for the simulated nonisothermal data (*n* = 3). Numbers by the symbols are heating rates in K min^−1^.

Equation (4) was employed to convert all simulated data into the α vs. *t* form. Then, the resulting data were used to construct the ln[−ln(1 − *α*)] vs. ln*t* plots for the α range 0.05–0.95, as usually done in practical analyses. The plots are shown in Figure 2. Although they are slightly nonlinear, they are fitted well with a straight line. All respective coefficients of correlation exceed 0.998 and are statistically significant. Regardless of the heating rate, the slope of all plots is ~7.5. This result clearly confirms the theoretical conclusion that the slope of the respective plots does not equal the Avrami exponent, which has to be 3. If one is to interpret this slope as *n*, the value exceeds significantly the true one, as was found in the tests by Zhang et al. [6] and Kourtidou and Chrissafis [7].

**Figure 3:**
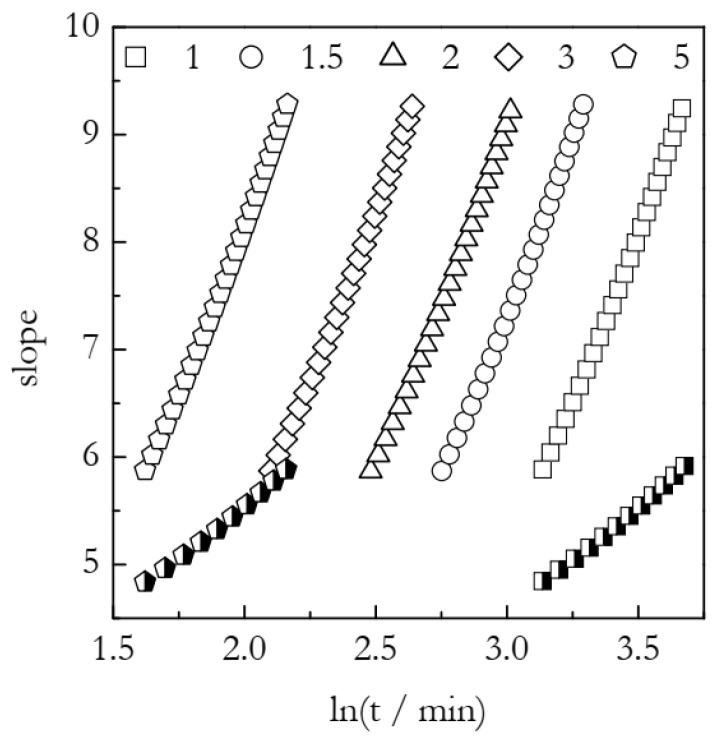
Theoretical slope values estimated for the simulated nonisothermal data. Open symbols for Equation (18), semi-solid symbols for Equation (9). Numbers by the symbols are heating rates in K min^−1^.

Furthermore, as suggested by Equation (18), the slope for the ln[−ln(1 − *α*)] vs. ln*t* plot is time dependent, i.e., the plot is nonlinear. However, Figure 2 presents straight line fits that naturally have a constant slope. By its meaning, such a slope is a value averaged over the corresponding time region. The actual variable slopes calculated in accord with Equation (18) are depicted in Figure 3. It is seen that the theoretical values of the slope vary approximately between six and nine. Thus, the respective average value is about 7.5. This is the slope value determined from the linear fits in Figure 2, which confirms the validity of Equation (18).

In addition, Figure 3 presents the slopes predicted by Equation (9). As stated above, this equation derives from Equation (6), which is deemed invalid so that Equation (6) should not be expected to predict the correct values of the slope. Indeed, the predicted value varies between five and six. That is, the average is around 5.5, which is markedly smaller than the 7.5 obtained directly from linear fits of the ln[−ln(1 − *α*)] vs. ln*t* plots (Figure 2). Therefore, Equation (9) inherits the invalidity of Equation (6).

**Figure 4:**
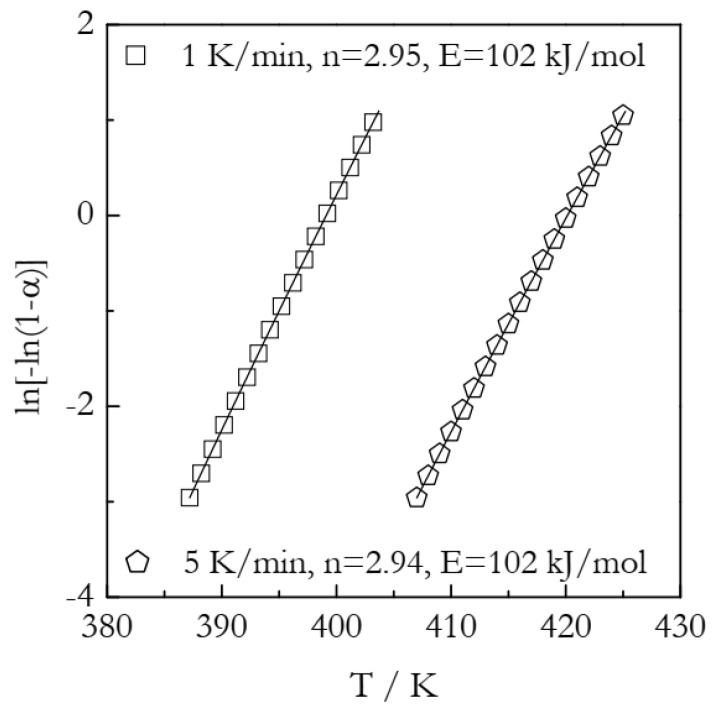
Simulated data fitted by Equation (16).

Although the results of the present study demonstrate that the ln[−ln(1 − *α*)] vs. ln*t* plots are useless for the purpose of the Avrami analysis of nonisothermal data, there appears to be a possibility of such analysis with the aid of Equation (16) that predicts a nonlinear dependence of ln[−ln(1 − *α*)] vs. *T*. In other words, one can use experimental data to construct such a plot and then fit Equation (16) to it. As a result, the fit parameter in the second term can be used to determine the Avrami exponent. Then, substitution of the resulting *n* into the fit parameter in the third term should permit the determining of the activation energy. This type of analysis was tried on the simulated data. The results are displayed in Figure 4. The fits are practically perfect (the coefficient of correlation is 1.0). However, the obtained value of the Avrami exponent is 2.94–2.95 which is 2% smaller than the true value of 3.00. This deviation probably arises from the approximate nature of Equation (16). Of course, the substitution of the underestimated *n* value gives rise to an overestimated value of *E*. The latter is 2% larger than the true value of 100 kJ mol^−1^. It should be noted that the obtained value of the Avrami exponent is noticeably less accurate than the one estimated by the Ozawa method from the same simulated data. The Ozawa method consistently produced *n* values accurate to the third decimal place, i.e., 3.000 [36]. With this in mind, Equation (16) may be worth looking into as a means of obtaining a quick and approximate estimate for *n*. On the other hand, the resulting estimate is incomparably more accurate than the one produced by the Jeziorny method.

## 6. Conclusions

The theoretical analysis demonstrates that the Jeziorny method is a faulty approach to the Avrami analysis of nonisothermal crystallization. The method constructs the experimental plots of ln[−ln(1 − *α*)] vs. ln*t* and fits them with a straight line. It is shown that the slope of the resulting line does not equal the Avrami exponent and the intercept the Avrami rate constant. The latter is impossible to determine either with or without the “correction”, which is explained to be meaningless. A theoretical equation that evaluates the slope of the ln[−ln(1 − *α*)] vs. ln*t* plot has been derived. It indicates that the plot is nonlinear and its slope varies rather widely with time so that when fitted with a straight line it yields an averaged value that tends to markedly exceed the Avrami exponent. Overall, the Jeziorny method either with or without the “correction” appears rather useless in the Avrami analysis of nonisothermal crystallization and, thus, should be avoided. The same conclusion applies to other techniques that substitute nonisothermal data into the isothermal Avrami plot.

## Data Availability

Not applicable.

## References

[1] Schultz J.M. (2001). Polymer Crystallization.

[2] Mandelkern L. (2004). Crystallization of Polymers.

[3] Ozawa T. (1971). Kinetics of nonisothermal crystallization. Polymer.

[4] Scopus.com.

[5] Jeziorny A. (1978). Parameters characterizing the kinetics of the non-isothermal crystallization of poly(ethylene terephthalate) determined by d.s.c. Polymer.

[6] Zhang Z., Xiao C., Dong Z. (2007). Comparison of the Ozawa and modified Avrami models of polymer crystallization under nonisothermal conditions using a computer simulation method. Thermochim. Acta.

[7] Kourtidou D., Chrissafis K. (2021). Nonisothermal Crystallization Kinetics: Studying the Validity of Different Johnson–Mehl–Avrami–Erofeev–Kolmogorov (JMAEK) Based Equations. Thermochim. Acta.

[8] Qu D., Gao H., Wang Q., Bai Y., Li N. (2020). Non-isothermal crystallization kinetics of bio-based poly(butylene-co-isosorbide succinate) (PBIS). J. Therm. Anal. Calorim..

[9] Nouira S., Hassine T., Fitoussi J., Shirinbayan M., Gamaoun F., Tcharkhtchi A. (2022). Non-isothermal crystallization kinetics and its effect on the mechanical properties of homopolymer isotactic polypropylene. J. Polym. Res..

[10] Lou S., Zhang H., Liu F., Yin W., Ren G., Chen Z., Su C. (2022). Effects of post-treatment on crystallization behavior of glass fiber-reinforced polyamide 66 composite with red phosphorus flame retardant. J. Therm. Anal. Calorim..

[11] Nakamura K., Watanabe T., Katayama K., Amano T. (1972). Some Aspects of Nonisothermal Crystallization of Polymers. I. Relationship Between Crystallization Temperature, Crystallinity, and Cooling Conditions. J. Appl. Polym. Sci..

[12] Piorkowska E., Galeski A., Haudin J.-M. (2006). Critical assessment of overall crystallization kinetics theories and predictions. Prog. Polym. Sci..

[13] Vyazovkin S. (2018). Nonisothermal crystallization of polymers: Getting more out of kinetic analysis of differential scanning calorimetry data. Polym. Cryst..

[14] Vyazovkin S. (2020). Activation energies and temperature dependencies of the rates of crystallization and melting of polymers. Polymers.

[15] Cebe P., Hong S.D. (1986). Crystallization behaviour of poly(etherether-ketone). Polymer.

[16] Wang L., Zhang F., Bai Y., Ding L. (2016). Non-isothermal melt-crystallization kinetics of poly (ethyleneterephthalate-co-sodium-5-sulfo-iso-phthalate). Thermochim. Acta.

[17] Jhu Y.S., Yang T.C., Hung K.C., Xu J.W., Wu T.L., Wu J.H. (2019). Nonisothermal Crystallization Kinetics of Acetylated Bamboo Fiber-Reinforced Polypropylene Composites. Polymers.

[18] Miyagawa Y., Adachi S. (2019). Analysis of Nonisothermal Crystallization of Rapeseed Oil by Deconvolution of Differential Scanning Calorimetry Curve. J. Oleo Sci..

[19] Chen W.M., Yang M.C., Hong S.G., Hsieh Y.S. (2019). Effect of soft segment content of Pebax® Rnew on the properties of Nylon-6/SMA/PEBA blends. J. Polym. Res..

[20] Gaonkar A.A., Murudkar V.V., Deshpande V.D. (2020). Comparison of crystallization kinetics of polyethylene terephthalate (PET) and reorganized PET. Thermochim. Acta.

[21] Li X., Zou M., Lei L., Xi L. (2021). Non-Isothermal Crystallization Kinetics of Poly(ethylene glycol) and Poly(ethylene glycol)-B-Poly(ε-caprolactone) by Flash DSC Analysis. Polymers.

[22] de Bruijn T.J.W., de Jong W.A., van den Berg P.J. (1981). Kinetic parameters in Avrami-Erofeev type reactions from isothermal and non-isothermal experiments. Thermochim. Acta.

[23] Yinnon H., Uhlmann D.R. (1983). Applications of thermoanalytical techniques to the study of crystallization kinetics in glass-forming liquids, Part I: Theory. J. Non-Cryst. Solids.

[24] Fatemi N., Whitehead R., Price D., Dollimore D. (1986). Some comments on the use of Avrami-Erofeev expressions and solid state decomposition rate constants. Thermochim. Acta.

[25] Khanna Y.P., Taylor T.J. (1988). Comments and Recommendations on the Use of the Avrami Equation for Physico-Chemical Kinetics. Polym. Eng. Sci..

[26] Brown M.E., Galwey A.K. (1989). Arrhenius Parameters for Solid-State Reactions from Isothermal Rate-Time Curves. Anal. Chem..

[27] Henderson D.W. (1979). Experimental analysis of nonisotermal transformations involving nucleation and growth. J. Therm. Anal..

[28] Henderson D.W. (1979). Thermal analysis of non-isothermal crystallization kinetic in glass forming liquids. J. Non-Cryst. Solids.

[29] Farjas J., Roura P. (2006). Modification of the Kolmogorov–Johnson–Mehl–Avrami rate equation for non-isothermal experiments and its analytical solution. Acta Mater..

[30] Lauritzen J.I., Hoffman J.D. (1973). Extension of theory of growth of chainfolded polymer crystals to large undercoolings. J. Appl. Phys..

[31] Starink M.J. (2007). Activation energy determination for linear heating experiments: Deviations due to neglecting the low temperature end of the temperature integral. J. Mater. Sci..

[32] Flynn J.H. (1997). The ‘Temperature Integral’—Its use and abuse. Thermochim. Acta.

[33] Starink M.J. (2003). The determination of activation energy from linear heating rate experiments: A comparison of the accuracy of isoconversion methods. Thermochim. Acta.

[34] Vyazovkin S., Dranca I. (2006). Isoconversional Analysis of Combined Melt and Glass Crystallization Data. Macromol. Chem. Phys..

[35] Papageorgiou G.Z., Bikiaris D.N., Achilias D.S. (2007). Effect of molecular weight on the cold-crystallization, of biodegradable poly(ethylene succinate). Thermochim. Acta.

[36] Vyazovkin S., Galukhin A. (2022). Problems with Applying the Ozawa–Avrami Crystallization Model to Non-Isothermal Crosslinking Polymerization. Polymers.

